# Clinical Comparison of MEBO and Hyaluronic Acid Gel in the Management of Pain after Free Gingival Graft Harvesting: A Randomized Clinical Trial

**DOI:** 10.1155/2021/2548665

**Published:** 2021-08-14

**Authors:** Ahmed Hassan, Enji Ahmed, Dalia Ghalwash, Azza Ezz Elarab

**Affiliations:** ^1^Oral Medicine and Periodontology Department, Faculty of Dentistry, Cairo University, Postal Code: 11553, Cairo, Egypt; ^2^Oral Medicine and Periodontology Department, Faculty of Dentistry, The British University in Egypt, Postal Code: 11837, El- Sherouk, Egypt

## Abstract

**Aim:**

This study aimed to compare the effect of MEBO versus 0.2% hyaluronic acid gel (Gengigel^®^) applied to the palatal donor site on postoperative pain reduction and wound healing after free gingival graft harvesting. *Methodology*. Thirty-nine patients with different mucogingival defects were included in this study for harvesting a free gingival graft (FGG) for soft tissue augmentation. Patients were randomly assigned into three equal groups: group I (MEBO), group II (0.2% HA Gengigel^®^), and group III (control). Postoperative pain was recorded by using the Visual Analogue Scale (VAS). Analgesic consumption was recorded for 7 days postoperatively. Wound size was measured at baseline and on days 3, 7, 14, and 21. Postoperative healing of the palatal wound was assessed by the VAS score for color match on days 3, 7, 14, 21, and 42.

**Results:**

Results of this study showed significant postoperative pain reduction for the three groups; MEBO showed statistically significant less VAS score compared to the other two groups, while HA showed statistically significant less VAS score compared to the control group. Both MEBO and HA showed statistically significant less total analgesic consumption. No statistically significant difference was observed between groups for wound size. MEBO showed statistically significant higher VAS for color match.

**Conclusions:**

Both treatment modalities could reduce postoperative pain following harvesting of FGG and improve the palatal wound healing. However, MEBO showed better outcomes in terms of postoperative pain and color match.

## 1. Introduction

Surgical treatment of mucogingival defects aims at obtaining soft tissue coverage of exposed root surfaces and/or augmentation of gingival tissue dimensions. Autogenous gingival grafts are still considered the gold-standard procedure in both single and multiple root coverage with proven clinical success [1, 2], with the palate being the most frequent donor site for grafts [3].

A free gingival graft is considered to be the most reliable method for increasing the attached gingiva and is one of the most frequently used techniques [4]. This treatment modality is used to increase the keratinized gingiva, prevent and treat gingival recession, improve esthetics, reduce or eliminate root hypersensitivity, increase the vestibular depth, and ameliorate pigmented and pathologic oral mucosa [5].

A whole free gingival graft (FGG) may be used at the recipient site or the graft may be deepithelialized after being harvested from the palate to get a subepithelial connective tissue graft (SCTG) [6]. Both techniques can be used to increase tissue thickness, to cover gingival recession, and/or to prevent the development and progression of gingival recession [2].

The palate is the most common donor site for gingival grafts [7]. FGG is considered superior to synthetic or allogenic grafts owing to its autogenous nature, which yield them excellent clinical results [8]. However, harvesting a FGG leaves an open wound at the donor site, which takes two to four weeks to heal. Patients' postoperative pain and discomfort and the delayed wound-healing in the palate are of great concern [4].

Recently, patients' subjective assessment of medical procedures has gained importance in healthcare; thus patients' expectations might be crucial in the selection of treatment measures [7].

The Moist Exposed Burn Ointment (MEBO) is a Chinese burn ointment with a registered USA patented formulation since 1995. MEBO is a pure herbal extract, which is natural in origin, containing beta-sitosterol, *Phellodendron amurense*, *Scutellaria baicalensis*, *Coptis chinensis*, *Pheretima aspergillum*, Beeswax, and sesame oil [9]. MEBO was claimed to have analgesic and antibacterial effect, while its beta-sitosterol component possesses an anti-inflammatory effect [10]. Sesame oil extract has a beneficial role in wound-healing, and its wound-healing properties may be due to the free radical scavenging and its antioxidant capacity [11].

0.2% hyaluronic acid (HA) gel was known for hygroscopicity that allows it to maintain conformational stiffness and to retain water [12]. Another major feature is viscoelasticity that provides stability and elasticity to tissues and delays penetration of viruses and bacteria [13]. In periodontology, HA has been advocated as monotherapy [14] or as an adjunct to nonsurgical and/or surgical periodontal treatment to reduce inflammation and promote wound-healing [15].

Both MEBO [16] and HA [12] have shown promising results in promoting healing and postoperative pain reduction of intraoral wounds that heal by secondary intention. Therefore, the aim of this study was to compare the effect of MEBO versus 0.2% hyaluronic acid gel (Gengigel^®^) applied to palatal donor site on postoperative pain reduction and wound-healing after free gingival graft harvesting.

## 2. Subjects and Methods

This study was conducted in the Oral Medicine and Periodontology Department, Faculty of Dentistry, Cairo University, Egypt. Thirty-nine subjects were selected from the outpatient clinic, Oral Medicine and Periodontology Department, Faculty of Dentistry, Cairo University, between March 2019 and September 2020. This clinical trial was registered in U.S. National Institutes of Health Clinical Trials Registry (Clinicaltrials.gov ID: NCT03728244).

The study protocol was approved by the Ethics Committee of Scientific Research, Faculty of Dentistry, Cairo University, on 25 December 2018 (approval number: 18-12-17). Sample size calculation was achieved using PS program (Power and Sample Size program: biostat.mc.vanderbilt.edu/twiki/bin/view/Main/PowerSampleSize) and approved by medical biostatistics unit, Cairo University, in October 2018.

Inclusion criteria included the following: patients with mucogingival defects scheduled for free gingival graft, systemically healthy patients, and patients with good oral hygiene.

Exclusion criteria included the following: patients with any uncontrolled local or systemic disease where periodontal plastic surgery might be contraindicated, history of recent periodontal surgery at the donor site, smokers, pregnancy and lactation, patients allergic to the used agents, severe gagging reflex, and inability or unwillingness to provide informed consent.

Patients were randomly assigned with simple randomization using computer-generated random numbers done by the cosupervisor E. A. into three equal groups; in group I, MEBO was applied to the palatal donor site and covered with periodontal pack; in group II, 0.2% hyaluronic acid gel (Gengigel^®^) was applied to the palatal donor site and covered with periodontal pack; and, in group III (control group), palatal donor site was covered with periodontal pack only.

The palatal donor site in the three groups was prepared, and then the decision of which drug to be used to protect the denuded area was made according to the randomized numbers in a sequentially numbered, opaque sealed envelope. The number was picked by the cosupervisor D. G.

## 3. Treatment Protocol

### 3.1. Presurgical Phase

Full-mouth supragingival and subgingival debridement was performed using ultrasonic device and Gracey's curettes. Proper oral hygiene instructions were given to the patients including brushing teeth 2 times daily by soft toothbrush.

Chemical plaque control with 0.125% Chlorhexidine HCL mouthwash was prescribed to be used twice daily for 2 weeks.

### 3.2. Preparation of the Recipient Site

In the three groups, preparation of the recipient site was performed according to Zucchelli and Mounssif [17] as follows: Two horizontal incisions were performed, being traced 1 mm coronal to the cementoenamel junction (CEJ) and extending 3 mm mesiodistally. Two vertical incisions diverging slightly in a coronoapical direction and extending 4 to 5 mm beyond mucogingival junction were performed. The trapezoidal area thus outlined was dissected using split-thickness incision to expose 3 to 4 mm of the apical periosteum to bone dehiscence. The soft tissue consisting mainly of alveolar mucosa that covered the recipient bed was removed with surgical scissors.

## 4. Harvesting the Free Gingival Graft (FGG) from the Palate

In all of the three groups, FGG was harvested from the palate as described by Zucchelli et al. [3] as follows: Two horizontal incisions were performed, where the coronal incision was 2 mm apical to the gingival margin of the adjacent teeth and two vertical incisions were traced to delineate the area to be grafted. The blade was inserted along the coronal horizontal incision at one edge perpendicular to the bone and once the adequate thickness of the graft was obtained which was 1–1.5 mm, the direction of the blade was changed to be parallel to the hard palate and moved in mesiodistal direction elevating the graft at one side until the graft became completely detached from the palate. The thickness of the graft was maintained uniform while proceeding apically with the blade. Care was taken to avoid removing the palatal periosteum. Once the graft was harvested, it was placed on a sterile gauze with saline to avoid the shrinkage of the graft. The FGG was positioned and firmly adapted to the recipient area and stabilized with two simple interrupted periosteal sutures and a criss-cross sling suture using 5–0 Vicryl suture material (M-Nature sutures; International Sutures Manufacturing Co., Egypt.) and Castroviejo Needle Holder (Nordent Manufacturing Inc., USA) [17].

## 5. Management of the Palatal Wound

After harvesting the FGG, the bleeding was controlled by application of pressure using sterile gauze for 5 min until it stopped.

### 5.1. Group I (MEBO)

MEBO (Julphar^®^, Ras Al Khaimah, United Arab Emirates) was applied using sterile plastic syringe, and the palatal wound was immediately covered by noneugenol periodontal pack (Pericem) (Pericem cement quengco, Non-Eugenol, Italy). Three days postoperatively, the patients were recalled, and the periodontal pack was removed for evaluating the healing of the palatal wound. Then MEBO was reapplied again, and the palatal donor site was repacked with periodontal pack.

### 5.2. Group II (0.2% HA)

0.2% hyaluronic acid gel (Gengigel^®^, Ricerfarma S.r.l., Milano, Italy) was applied using sterile plastic syringe and the palatal wound was immediately covered by noneugenol periodontal pack (Pericem). Three days postoperatively, the patients were recalled, and the periodontal pack was removed for evaluating the healing of the palatal wound. Then 0.2% HA gel was reapplied again, and the palatal donor site was repacked with periodontal pack.

### 5.3. Group III (Control Group)

The palatal wound was immediately covered by noneugenol periodontal pack (Pericem). Three days postoperatively, the patients were recalled, and the periodontal pack was removed for evaluating the healing of the palatal wound and then repacked with periodontal pack.

### 5.4. Postsurgical Protocol

**Figure 1:**
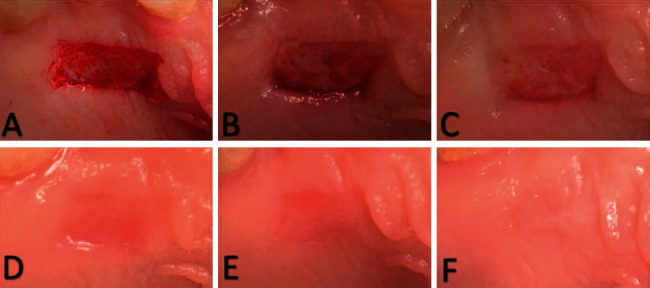
Clinical photographs showing palatal wound healing for the MEBO group: (a) day 0, (b) day 3, (c) day 7, (d) day 14, (e) day 21, and (f) day 42.

**Figure 2:**
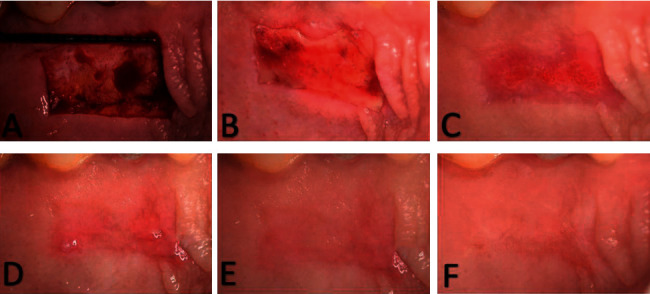
Clinical photographs showing palatal wound healing for the HA group: (a) day 0, (b) day 3, (c) day 7, (d) day 14, (e) day 21, and (f) day 42.

The periodontal pack was finally removed after 1 week [3]. Patients received 600 mg Ibuprofen (Abbott, Egypt) on surgery day for pain control, and they were instructed to take Ibuprofen 600 mg only if needed and to count the number of pills taken for the purpose of indirect pain measurement via mean consumption of analgesics (mg). Patients were advised to rinse with antiseptic mouth rinse (0.12% Chlorhexidine HCL) twice daily for 1 minute for a period of two weeks after the surgery. Patients were instructed to avoid any hard brushing and trauma to the surgical site for 3 weeks. Three weeks postsurgically, the patients were instructed to gently brush the operated area with a soft toothbrush using circular scrub technique. The sutures were removed fourteen days after the surgery from the recipient site that was augmented by the FGG. Clinical photographs were taken for the palatal wound on day 3, then after 1, 2, and 3 weeks, and finally on day 42 postsurgically for evaluation of the healing of the palatal wound at different time intervals; see Figures 1 and 2.

### 5.5. Primary Outcome

#### 5.5.1. Postoperative Pain (Visual Analogue Scale)

Pain score was reported by the patient directly through Visual Analogue Scale (VAS) score (between 0 and 10; 0: no pain, 1: minimal pain, 5: moderate pain, and 10: severe pain) [18]. VAS was recorded daily for 1 week [7].

#### 5.5.2. Postoperative Pain (Mean Consumption of Analgesics)

Postoperative pain was assessed indirectly by mean consumption of analgesics for 7 days postoperatively, recorded in milligrams [3].

### 5.6. Secondary Outcomes

#### 5.6.1. Wound Size

Wound size was measured using (UNC-15) periodontal probe to the nearest measurement of 0.5 mm on surgery day [19]. Wound-tracing was done on days 3, 7, 14, and 21 via planimetric method, a transparent film was placed on top of the wound, and the margin of the wound was traced with a pen. The tracing was subsequently placed on a metric grid and wound area was determined by counting the number of squares in the grid covered by the traced area [20].

#### 5.6.2. Color Match

On day 3, day 7, day 14, day 21, and day 42, the color of the palatal mucosa was assessed by comparing it with that of the adjacent and opposite side by using the objective VAS (VAS score 0–10) represented by a continuous line by the main supervisor who was blinded to the treatment group assignment. A score of 0 indicates no color match, and a score of 10 indicates excellent color match with the adjacent tissues [12].

## 6. Results

### 6.1. Demographic Data

**Figure 3:**
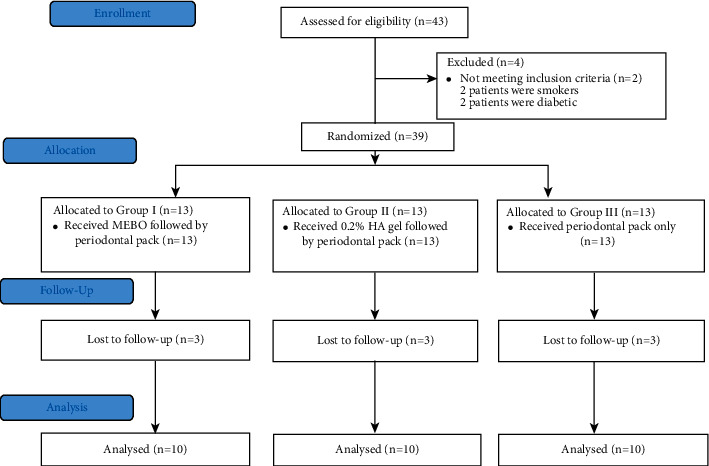
CONSORT flow diagram.

The study population in this randomized, controlled, parallel-grouped clinical trial included 39 patients with different mucogingival defects that required harvesting a free gingival graft (FGG). Only 30 patients completed their follow-up period. The data of all subjects who were examined and completed their follow-ups in the present study were recorded, tabulated, and subjected to statistical analysis. This study was reported according to CONSORT guidelines [21]; see Figure 3.

Females constituted 80% and males 20% of patients in group I, while in group II and group III 90% of patients were females and 10% were males, with no statistically significant difference between groups (*p*=0.749). Patient's age ranged from 24 to 49 years, and the mean ± SD age of group I was 40.60 ± 2.02, that of group II was 37.80 ± 2.65, and that of group III was 34.20 ± 2.19, with no statistically significant difference between groups (*p*=0.163).

### 6.2. Primary Outcome

#### 6.2.1. Postoperative Pain (Visual Analogue Scale (VAS))

Regarding median and range values for intragroup comparison in postoperative pain measured by VAS daily from day 1 to day 7 within each group, there was a statistically significant decrease in VAS by time in the three groups.

Median and range values of intergroup comparison in postoperative pain measured by VAS daily from day 1 to day 7 in the three groups are presented in Table 1. On days 1 and 2, there was no statistically significant difference between the three groups with the highest median and range for VAS pain score being recorded in group III. On day 3, there was a statistically significant difference between the three groups in pain score being statistically significantly lower in group I and statistically significantly higher in group III. From day 4 to day 6, there was no statistically significant difference between the three groups with the highest median and range for VAS pain score being recorded in group III. On day 7, all patients in the three groups reported no pain at all.

#### 6.2.2. Postoperative Pain (Analgesic Consumption in mg)

Median and range values for intragroup comparison in postoperative pain measured by analgesic consumption (mg) daily from day 1 to day 7 in the three groups showed that the three studied groups showed statistically significant decrease in analgesic consumption from day 1 to day 7.

Results for median and range values for intergroup comparison of postoperative pain measured by analgesic consumption (mg) daily from day 1 to day 7 in the three groups are shown in Table 2. On day 1, the highest median and range values for total analgesic consumption were recorded in group III; however, this difference did not reach the level of statistical significance. On days 2 and 3, there was a statistically significant difference in analgesic consumption between the three groups, where the highest values were recorded in group III and the lowest values were recorded in group I. On day 4 and day 5, there was no statistically significant difference in analgesic consumption between the three groups. On day 6 and day 7, all patients in the three groups reported no analgesic consumption at all.

For total analgesic consumption (in the 7 days) per patient|, statistically significantly higher median and range values were recorded in group III in comparison to group II and group I. Meanwhile, statistical significance was not found between group I and group II.

### 6.3. Secondary Outcomes

#### 6.3.1. Wound Size

Results for mean and SD values for intragroup comparison of wound size measured by UNC 15 probe and transparent paper (mm) on days 0, 3, 7, 14, and 21 within each group showed the following: In group I, there was no statistically significant difference between the time points; however, there was a statistically significant reduction from day 0 and day 3 to day 7. In group II, there was no significant difference between day 0, day 3, and day 7, while there was a significant reduction on day 14 and day 21 compared to day 0 and day 3. There was a statistically significant reduction in wound size from day 0 to day 21. In group III, there was a gradual statistically insignificant decrease in wound size by time with no significant reduction in wound size on day 21.

Results for mean and SD values for intergroup comparison of wound size (mm) measured by UNC 15 probe and transparent paper (mm) on days 0, 3, 7, 14, and 21 between the three studied groups are presented in Table 3.

On day 0 and day 3, the highest mean value was recorded in group II; however, the difference between groups did not reach the level of statistical significance. On day 7, the highest mean values were recorded in group I and group II, respectively; however, the difference between groups was not statistically significant. On day 14 and day 21, the highest mean value was recorded in group I; however, the difference between groups did not reach the level of statistical significance.

#### 6.3.2. Color Match (Visual Analogue Scale)

Results for median and range values for intragroup comparison of color match measured by Visual Analogue Scale score within each group at all time points showed the following: In group I, values recorded on day 3, day 7, and day 14 were significantly lower compared to the subsequent observations. On day 42, the median and range values of color match score showed a statistically significant increase by time compared to day 3 and values recorded on day 21 and day 42 were significantly higher compared to previous observations.

In group II, the median and range values of color match score showed a statistically significant increase by time with the lowest value being on day 3 and highest value being on day 42. In group III, the median and range values of color match score showed a statistically significant increase by time with the lowest value being on day 3 and the highest values being on days 21 and 42. There was a statistically significant increase in color match by time in all groups.

Results for median and range values for intergroup comparison of color match measured by Visual Analogue Scale score between the three studied groups at all time points are presented in Table 4.

On day 3, day 7, and day 14, the highest median and range values were recorded in groups I and II, respectively, compared to group III; however, there was no statistically significant difference between the three groups.

On day 21, higher median and range values were recorded in groups I and II, respectively, compared to group III. There was no statistically significant difference between group II and both group I and group III. However, there was a significant difference between group I and group III. On day 42, significantly higher median and range values were recorded in group I in comparison to groups II and III.

## 7. Discussion

Various clinical trials were carried out aiming to reduce postoperative patients' morbidity following soft tissue grafts harvesting; most of these trials achieved positive outcomes using different materials such as hyaluronic acid (HA) gel [12], ozone therapy [22], topical simvastatin gel [23], and advanced platelet-rich fibrin (A-PRF) [24].

The present clinical trial aims to compare the effect of MEBO versus 0.2% hyaluronic acid gel applied to palatal donor site on postoperative pain reduction and wound-healing after FGG harvesting. Two concentrations are available for HA gel, 0.2% and 0.8%; however, in this clinical trial, 0.2% HA gel was used, since it was found to be more superior than 0.8% regarding the acceleration of the palatal wound-healing as reported by [12]. In the present study, MEBO was applied topically at the palatal wound site because, unlike currently used topical products, MEBO produces the necessary moist environment for optimal healing. MEBO provides physiological moisture for optimized wound-healing and reepithelialization and is also easy to apply irrespective of site, extent, and local condition of the wound. MEBO is pure herbal extract, which is natural in origin; thus it is neither irritant nor toxic to the oral mucosa [9].

Hyaluronic acid (HA) was also the product of choice in the present study to cover the palatal wound, since it has unique physiochemical and biological properties, being anti-inflammatory, antiedematous, antioxidant, bacteriostatic, and capable of accelerating wound-healing, preventing scar formation, and enhancing tissue regeneration through its ability to retain a large amount of water. HA has been the focus of study in many clinical trials due to its feasibility in the form of either gel or spray; also it is a noninvasive method for the patient which is easy to use. Therefore, HA was regarded as a useful dressing for treatment of the palatal wound following the harvesting of FGG [12, 25].

Various clinical trials have been done to test the efficacy of HA gel application in periodontal surgery [12, 14, 15]. However, very limited trials are available to test the efficacy of MEBO in periodontal surgery and intraoral wound-healing [16]. The present study was conducted on thirty-nine patients with different mucogingival problems indicated for FGG harvesting. The study ended with thirty patients, as seven patients did not complete their follow-ups due to Covid-19 pandemic and two patients did not attend their follow-ups for unknown reasons.

Postoperative pain is correlated more to wound depth rather than surface area [26]. Thus, in the present study, care was taken to obtain a uniform thickness of the graft to be ranged from 1 to 1.5 mm, as a thin graft was found to have a low resistance to functional forces and was more prone to secondary contraction. However, a more than 1.5 mm thick graft could provide more functional resistance to the forces but is more liable to the primary shrinkage [2], as well as to avoid exaggerated pain and necrosis encountered with graft thicker than 2 mm [26]. On the contrary, Wyrebek et al. [7] conducted a clinical trial on the effect of graft dimension on patients' morbidity and concluded that the correlation between postoperative morbidity and the graft size loses value when the palatal wound is covered by an individual stent.

Postoperative pain was measured in this study both directly via VAS and indirectly via consumption of analgesics; thus patients received Ibuprofen 600 mg on surgery day for pain control and were instructed to take Ibuprofen 600 mg only if needed to make sure that any reported pain scores are related to the intervention used [3]. No other systemic medications were prescribed including antibiotics. The prevalence of postoperative infections following periodontal surgery is less than 1% and this low risk does not justify the use of systemic antimicrobials just to prevent infections [27].

Reapplication of MEBO, HA, and periodontal pack was done on the third day. Although wound-healing can be traumatized during the removal and renewing of periodontal dressing, this was done on purpose for many reasons, on one side, to evaluate the wound at least at one measurement point as early as suggested by the relevant literature [26] and, on the other side, to use the periodontal dressing benefits on surgical wound care, wound protection, and pain reduction as well as HA and MEBO retention and renewal. Since periodontal dressings were reapplied to all groups, all patients encountered the same degree of trauma without jeopardizing the results of this study [12].

Postoperative pain was evaluated in this study daily till the seventh day as pain reaches its maximum level at the initial healing phase (0–3 days) and completely disappears on day 14 using 0.2% HA gel, even when compared to negative control [12]. Furthermore, Mahmoud et al. [16] evaluated postoperative pain using VAS for seven days only when comparing the effects of HA and MEBO to negative control on wound-healing in vestibular deepening procedures.

Wound size was measured in the present study via planimetric method of wound size assessment as using linear measurements only (length *x* width) does not provide accuracy due to irregular healing pattern of the palatal wound. This parameter was evaluated in the present study on day 0, day 3, day 7, day 14, and day 21 as complete epithelial healing occurs after 21 days [28].

Color match (CM) of the palatal wound was assessed by comparing it with that of the adjacent and opposite side by using the objective VAS. As healing of FGG takes place by secondary intention, color change is observed clearly and matching with adjacent normal tissue would reflect the degree of reepithelization and wound-healing. CM was evaluated in the present study on day 3, day 7, day 14, day 21, and day 42 [12, 29].

Results of the present study showed a statistically significant reduction by time in postoperative pain measured by VAS in group I (MEBO) since day 1 and showed significantly decreased postoperative pain to reach a VAS median of 0 (0–3) and median consumption of analgesics of 0 (0–0) on day 2 postsurgically with complete pain disappearance on day 6 for all subjects. These results are slightly superior to those of a study conducted by Mahmoud et al. [16] to evaluate the effect of MEBO on wound-healing in vestibular deepening procedures which reported a VAS median of 3 (2–3) on day 2 postsurgically. Vestibular deepening heals by secondary intention, which shows a similar pattern to palatal wound-healing. The previous study is the only study found in literature which uses MEBO to accelerate healing of postsurgical intraoral wounds that heal by secondary intention.

The results of the present study are in accordance with a previous study that used MEBO in treatment of upper gastrointestinal injuries which concluded that MEBO stimulates healing by improving microcirculation, promoting granulation tissue growth, and providing physiologic environment for wound repair by inhibiting bacterial overgrowth and providing a protective membrane preventing bacterial invasion [30].

For group II, results of the present study showed that 0.2% HA gel significantly reduced postoperative pain to reach a VAS median of 0 (0–5) and median consumption of analgesics on day 4 postsurgically with complete pain disappearance for all subjects on day 6, which could be explained by the anti-inflammatory, antiedematous, antioxidant, and antibacterial effects of HA [25]. The reduction of postoperative pain in the present study was more than that reported in another study in which VAS median was reported to be 2 (1–3) on day 4 [16]; this could be attributed to HA reapplication on day 3, while it was applied only once on surgery day in that study which could prolong the anti-inflammatory and antiedematous action of HA gel. On the other hand, our VAS median on day 3 was slightly higher but on day 7 the results of the present study were consistent with those of the study conducted by Yıldırım et al. [12].

For group III, results of the present study showed that the use of periodontal pack only could significantly reduce pain in terms of VAS and analgesic consumptions between day 1 and day 4 with complete disappearance of postoperative pain for all subjects on day 7, which is consistent with both related studies [12, 16].

Results of the present study showed a statistically significant reduction in postoperative pain measured by VAS in group I compared to group II and group III on day 2 and day 3. However, on day 3, group II also showed a statistically significant postoperative pain reduction when compared to group III. For intergroup comparison between MEBO and HA, no significant difference in terms of VAS score was present on day 2 and day 4, which is consistent with the results of the previous study [16], where no significant difference could be found on day 2 and day 4. However, in the present study, a significant difference in terms of pain reduction (VAS) in favor of group I was encountered when compared to group II on day 3. Because the pain perception is a subjective and personal feeling, VAS scale results may vary from a study to another [29].

Regarding indirect measurement of postoperative pain via analgesic consumption, the three studied groups showed a statistically significant decrease in their use from day 1 to day 7. However, in group I, median of analgesic consumption reached 0 on day 2, while group II and group III reached a median of 0 on day 4, reflecting the powerful analgesic and anti-inflammatory effect of MEBO.

All groups showed significant reduction in wound size from day 0 to day 42; however, no significant difference between groups was detected in wound size in each time point or in percent change in each time interval. These findings could be justified as the added effect of MEBO/HA is due to their anti-inflammatory properties of the active components rather than accelerating the epithelial creeping of palatal wound.

Concerning color match in the present study, all groups showed statistically significant increase by time. These results were consistent with the findings of Yıldırım et al. [12] for both 0.2% HA gel group and control group. A statistically significant difference between groups was observed on day 21 and day 42. Group I showed higher CM compared to group II on day 21 with no statistically significant difference but statistically significant higher CM compared to group III. A statistically significant higher value for CM was detected in group I on day 42 when compared to groups II and III.

Group II showed higher CM on day 21 when compared to group III but with no statistically significant difference, while the difference was statistically significant on day 42. This may be interpreted as a sign of better wound-healing which enables faster course of CM. Clinical studies have found that MEBO promotes debridement and epithelial repair and is associated with better scar quality [9, 30]. Furthermore, HA leads to tissue maturation and rapid epithelization, which ensures good CM [31].

To our knowledge, no study that reviewed the effect of MEBO on intraoral wound-healing used color match as an outcome. The pain reduction and rapid healing in intervention groups (group I and II) are possibly due to the anti-inflammatory and antioxidant effects of their main components as b-sitosterol for MEBO and sodium hyaluronate for HA gel rather than rapid epithelialization. This was reflected by our results, which showed improved outcomes for intervention groups in postoperative pain, analgesic consumption, and color match when compared to control group, while there was no statistically significant difference between groups in terms of wound size. However, MEBO showed superior outcomes in terms of postoperative pain control and color match compared to control and HA groups.

## 8. Conclusions

Topical application of MEBO or 0.2% HA gel (Gengigel^®^) on palatal wound following FGG harvesting could significantly reduce postoperative pain and promote palatal wound-healing. Nevertheless, MEBO application produced even more favorable effects on pain reduction and better color match which reflects the healing process. MEBO could be considered a practical alternative, since it is cheap, available, and easily applied.

## Figures and Tables

**Table 1:** Median and range values for intergroup comparison of postoperative pain measured by the VAS score daily from day 1 to day 7 in the three groups.

VAS pain score		Median	Min	Max	*p* value
Day 1	Group I	3	0.00	5.00	0.119 ns
	Group II	3	0.00	4.00	
	Group III	5	0.00	10.00	

Day 2	Group I	0^b^	0.00	3.00	0.006^*∗*^
	Group II	2.5^a^	0.00	5.00	
	Group III	4.5^a^	0.00	6.00	

Day 3	Group I	0^c^	0.00	1.00	0.008^*∗*^
	Group II	0.5^b^	0.00	4.00	
	Group III	4.5^a^	0.00	7.00	

Day 4	Group I	0	0.00	4.00	0.091 ns
	Group II	0	0.00	5.00	
	Group III	2	0.00	5.00	

Day 5	Group I	0	0.00	3.00	0.485 ns
	Group II	0	0.00	4.00	
	Group III	0	0.00	6.00	

Day 6	Group I	0	0.00	1.00	0.573 ns
	Group II	0	0.00	0.00	
	Group III	0	0.00	2.00	

Day 7	Group I	0	0.00	0.00	1 ns
	Group II	0	0.00	0.00	
	Group III	0	0.00	0.00	

Significance level: *p* ≤ 0.05; ^*∗*^significant; ns = nonsignificant.

**Table 2:** Median and range values for intergroup comparison of postoperative pain measured by analgesic consumption (mg) daily from day 1 to day 7 in the three groups.

Total analgesic consumption		Median	Min	Max	*p* value
Day 1	Group I	600	0.00	1200.00	0.068 ns
	Group II	600	0.00	1800.00	
	Group III	1500	0.00	1800.00	

Day 2	Group I	00^c^	0.00	0.00	0.002^*∗*^
	Group II	300^b^	0.00	1200.00	
	Group III	900^a^	0.00	1800.00	

Day 3	Group I	00^c^	0.00	0.00	0.004^*∗*^
	Group II	300^b^	0.00	1800.00	
	Group III	900^a^	0.00	1800.00	

Day 4	Group I	0.00	0.00	1800.00	0.415 ns
	Group II	0.00	0.00	1200.00	
	Group III	0.00	0.00	1200.00	

Day 5	Group I	0.00	0.00	600.00	0.573 ns
	Group II	0.00	0.00	0.00	
	Group III	0.00	0.00	1200.00	

Day 6	Group I	0.00	0.00	0.00	1 ns
	Group II	0.00	0.00	0.00	
	Group III	0.00	0.00	0.00	

Day 7	Group I	0.00	0.00	0.00	1 ns
	Group II	0.00	0.00	0.00	
	Group III	0.00	0.00	0.00	

Total	Group I	600^b^	0.00	3000.00	0.002^*∗*^
	Group II	1200^b^	0.00	4800.00	
	Group III	4200^a^	600	6000.00	

Significance level: *p* ≤ 0.05; ^*∗*^significant; ns = nonsignificant.

**Table 3:** Mean and SD values for the intergroup comparison of wound size (mm) in the three studied groups at all time points.

Wound size		Mean	Std. dev.	Std. error	95% confidence interval for mean		Min	Max	*p* value
					Lower bound	Upper bound			
Day 0	Group I	128.00	30.84	9.75	105.94	150.06	70.00	150.00	0.787 ns
	Group II	132.90	27.32	8.64	113.36	152.44	75.00	160.00	
	Group III	122.50	40.71	12.87	93.38	151.62	50.00	180.00	

Day 3	Group I	120.90	31.24	9.88	98.56	143.24	67.00	150.00	0.639 ns
	Group II	131.20	26.53	8.39	112.22	150.18	75.00	160.00	
	Group III	117.00	43.18	13.65	86.11	147.89	47.00	180.00	

Day 7	Group I	109.90	31.49	9.96	87.38	132.42	60.00	147.00	0.928 ns
	Group II	109.50	33.12	10.47	85.81	133.19	52.00	148.00	
	Group III	104.50	39.11	12.37	76.52	132.48	45.00	159.00	

Day 14	Group I	97.80	33.12	10.47	74.11	121.49	52.00	146.00	0.859 ns
	Group II	90.20	26.71	8.45	71.09	109.31	48.00	130.00	
	Group III	91.30	38.75	12.25	63.58	119.02	36.00	140.00	

Day 21	Group I	81.40	33.50	10.59	57.44	105.36	35.00	130.00	0.778 ns
	Group II	72.30	20.19	6.39	57.85	86.75	44.00	100.00	
	Group III	79.40	34.26	10.83	54.89	103.91	28.00	130.00	

Significance level: *p* ≤ 0.05; ns = nonsignificant.

**Table 4:** Median and range values for the intergroup comparison of color match measured by the Visual Analogue Scale score between the three studied groups at all time points.

Color match		Median	Min	Max	*p* value
Day 3	Group I	3	2.00	5.00	0.128 ns
	Group II	3	2.00	4.00	
	Group III	2	0.00	4.00	

Day 7	Group I	5	0.00	5.00	0.095 ns
	Group II	4.5	3.00	7.00	
	Group III	4	4.00	5.00	

Day 14	Group I	6.5	1.00	5.00	0.054 ns
	Group II	6	1.00	7.00	
	Group III	5	4.00	8.00	

Day 21	Group I	8^a^	4.00	7.00	0.032^*∗*^
	Group II	7^a,b^	4.00	7.00	
	Group III	6.5^b^	4.00	8.00	

Day 42	Group I	10^a^	6.00	9.00	0.006^*∗*^
	Group II	8^b^	4.00	8.00	
	Group III	7.5^b^	5.00	8.00	

Significance level: *p* ≤ 0.05; ^*∗*^significant; ns = nonsignificant.

## Data Availability

The authors declare that all the data supporting the results of this study are readily available upon demand.
